# Improving Outcomes in People with Spinal Cord Injury: Encouraging Results from a Multidisciplinary Advanced Rehabilitation Pathway

**DOI:** 10.3390/brainsci14020140

**Published:** 2024-01-28

**Authors:** Maria Grazia Maggio, Mirjam Bonanno, Alfredo Manuli, Rocco Salvatore Calabrò

**Affiliations:** 1 IRCCS Centro Neurolesi Bonino-Pulejo, Cda Casazza, SS 113, 98123 Messina, Italy; mariagrazia.maggio@irccsme.it (M.G.M.); roccos.calabro@irccsme.it (R.S.C.); 2 A.O.U. Policlinico “G. Martino”, Via Consolare Valeria, 98124 Messina, Italy; manulialfredo@gmail.com

**Keywords:** spinal cord injury, neurorehabilitation, multidisciplinary approaches, innovative technologies

## Abstract

Spinal cord injury (SCI) consists of damage to any segment of the spinal cord extending to potential harm to nerves in the cauda equina. Rehabilitative efforts for SCI can involve conventional physiotherapy, innovative technologies, as well as cognitive treatment and psychological support. The aim of this study is to evaluate the feasibility of a dedicated, multidisciplinary, and integrated intervention path for SCI, encompassing both conventional and technological interventions, while observing their impact on cognitive, motor, and behavioral outcomes and the overall quality of life for individuals with SCI. Forty-two patients with SCI were included in the analysis utilizing electronic recovery system data. The treatment regimen included multidisciplinary rehabilitation approaches, such as traditional physiotherapy sessions, speech therapy, psychological support, robotic devices, advanced cognitive rehabilitation, and other interventions. Pre–post comparisons showed a significant improvement in lower limb function (Fugl Meyer Assessment-FMA < 0.001), global cognitive functioning (Montreal Cognitive Assessment-MoCA *p* < 0.001), and perceived quality of life at both a physical and mental level (Short Form-12-SF-12 *p* < 0.001). Furthermore, we found a significant reduction in depressive state (Beck Depression Inventory-BDI *p* < 0.001). In addition, we assessed patient satisfaction using the Short Form of the Patient Satisfaction Questionnaire (PSQ), offering insights into the subjective evaluation of the intervention. In conclusion, this retrospective study provides positive results in terms of improvements in motor function, cognitive functions, and quality of life, highlighting the importance of exploring multidisciplinary approaches.

## 1. Introduction

Spinal cord injury (SCI) is damage to any segment of the spinal cord, with a partial or complete interruption of signals from and to the brain [1]. Individuals with SCI not only show alterations in sensorimotor and autonomic functions but may also encounter mental, emotional, and social repercussions, adding complexity to the overall impact of such injuries [2]. Depression is one of the most common mental health problems in patients with SCI, affecting up to 40% of patients during rehabilitation [3]. The epidemiology of traumatic SCI is constantly changing, along with the types of associated SCI. The annual worldwide incidence of traumatic SCI ranges from 3.3 to 195.4 cases per million based on subnational studies [4]. 

The severity of SCI is variable [5]. Minimal nerve cell death can facilitate a near-complete recovery [6]. Conversely, complete injuries result in the loss of muscle control, feeling, or function [5,6]. Considering the pivotal role of the spinal cord in orchestrating our physical capabilities, timely and targeted interventions, including a specialized rehabilitation, become fundamental. Typically, rehabilitative efforts for SCI commonly involve conventional physiotherapy, emphasizing muscle strengthening exercises, static and dynamic balance activities, and gait training to avert complications associated with immobility, such as muscle atrophy, pressure ulcers, and autonomic nervous system decline [7]. Recent advances in neurorehabilitation, encompassing robotic devices and virtual reality (VR) systems, have been integrated to facilitate more intensive, repetitive, and task-oriented training for enhanced outcomes [8]. According to a systematic review [9], the application of VR in motor training for SCI patients holds promise in improving sensorimotor functions, motivation, and engagement. Specifically, VR is an advanced technology that immerses users in a simulated environment, allowing them to interact with virtual elements in an immersive and engaging way. This technology finds significant application in the field of motor and cognitive training, providing an innovative approach to improving the skills of individuals with SCI. VR offers different shades of “immersion” and “presence.” Immersion concerns the objective perceptual experience linked to the characteristics of the system and the virtual task (the physical sensation of being in a virtual world), while presence involves a more subjective aspect relating to the perceptions and characteristics of the user (involvement and activation resulting from activity) [10,11]. The devices can be classified, based on the degree of virtual immersion, into non-immersive (for example, Virtual Reality Rehabilitation System—VRRS Evo, Padua, Italy), semi-immersive (for example, Nirvana Bts Bioengineering, Milan, Italy), and immersive (for example, Computer Assisted Rehabilitation Environment—CAREN). Generally, semi-immersive and non-immersive VR tools use a screen to display the environment with a low level of immersion and presence. Otherwise, immersive systems provide full integration of the user into the virtual environment, incorporating gloves and head-mounted displays, which provide sensory input to the patient.

In the area of motor training, VR facilitates the creation of virtual scenarios that replicate real-life situations, allowing users to perform targeted exercises aimed at improving muscle strength, coordination, and mobility. For example, using devices such as the CAREN system, users can engage in interactive simulations of motor activities that require precise and timely responses, contributing to the development and maintenance of fundamental motor skills. CAREN represents an advanced immersive VR platform that integrates visual, auditory, and motor elements to create a complete virtual environment [12].

From a cognitive perspective, VR offers the opportunity to create stimulating environments that test users’ cognitive abilities. Through interacting with complex virtual scenarios, SCI patients can address cognitive challenges and improve memory, concentration, and problem-solving skills. BTS Nirvana is a semi-immersive VR system that integrates visual, auditory, and motor elements to create a complete virtual environment, thus engaging and stimulating cognitive functions. Leveraging motion sensors and real-time feedback, Nirvana allows users to participate in interactive simulations of everyday activities [13]. 

Therefore, VR offers an immersive and motivating experience, allowing users to face personalized motor challenges and monitor progress over time. The application of VR in motor and cognitive training for SCI patients not only provides a controlled and safe environment for practice but also offers a motivating and engaging experience. This technology opens new horizons in rehabilitation, allowing patients to face personalized challenges and the possibility of adapting exercises to the specific needs of each individual, maximizing the benefits of motor and cognitive training [14].

On the other hand, robotic devices, such as exoskeletons, are used to begin inpatient rehabilitation as soon as individuals with SCI are medically stable to tolerate upright position and/or partial or full weight-bearing. Verticalization in the very early stages can be carried out by using the robotic device ERIGO [15], while robotic-assisted gait training may be provided using different robots. Exoskeletons are defined as wearable devices able to reproduce passive and active-assisted movements with different degrees of assistance. Generally, exoskeletons are subcategorized into tethered (e.g., Lokomat-Pro, Hokoma, Switzerland), which can be combined with a VR screen to further engage the patient’s motivation, and untethered (e.g., Re-walk-ReWalk USA, Ekso-GT-Ekso Bionics USA, Indego-Ekso Bionics USA), which is used to promote over-ground gait [16,17,18] and improve non-motor symptoms, like constipation and pain. The former is also provided with a body weight support system that can unload the patient’s weight during walking so that complete SCI may also benefit from the training. Incomplete SCI patients may also be treated by means of end-effectors, i.e., robotic devices in which the movement is generated by the distal part of the body (i.e., foot for lower limb), giving the opportunity to safely train ascending and descending stairs [19]. 

Although SCI directly impacts movement and sensation but does not have a direct effect on cognition, a systematic review reveals that up to 64% of individuals with SCI exhibit some degree of cognitive impairment [20]. Indeed, in a case report and recent study [21,22], our group demonstrated the efficacy of training using a non-immersive VR system in individuals with SCI. This study provided evidence of benefits for both motor and cognitive outcomes [22,23]. Consequently, VR emerges as a valuable tool in promoting multi-sensory stimulation, engaging the cortical sensory–motor network and subcortical brain regions, thereby potentially enhancing motor and cognitive functions [16,17,22]. Thus, there arises the necessity to adopt an integrated approach that combines conventional rehabilitation methodologies with technological innovations, such as VR and robotics. This multidisciplinary treatment aims to provide a comprehensive approach for individuals affected by SCI. 

From this perspective, the purpose of the present study is to evaluate the feasibility of a dedicated, multidisciplinary, and integrated intervention path for SCI. Indeed, efficacy was not our outcome since we did not compare the innovation pathway to traditional approaches.

In particular, we sought to:

- Evaluate the feasibility of an integrated intervention path for SCI.
- Investigate how the intervention path influences the overall quality of life for individuals with SCI.
- Observe and measure the impact of the intervention path on cognitive, motor, and behavioral outcomes.

## 2. Materials and Methods

### 2.1. Study Design and Population

To determine the sample size for this study, we utilized a 95% confidence level and a 5% margin of error in G*Power. Based on these parameters and a power analysis, the minimum required sample size of 16 patients with Spinal Cord Injury (SCI) was calculated with a statistical power of 80% (4, 7, 9, 17). Subsequently, data from forty-two patients with SCI who attended our Robotic and Behavioral Neurorehabilitation (NR) Unit between January 2018 and February 2020 were included in the analysis, utilizing information from the electronic recovery system.

This retrospective study adhered to the principles of the amended version of the Declaration of Helsinki, 2013 [24]. Prior to entering the rehabilitation pathway, all subjects provided written informed consent for the use of their data in research. This study’s retrospective nature and data extraction from an electronic medical record aimed to minimize scoring bias. Inclusion criteria were as follows: (i) age ≥ 18 years; (ii) diagnosis of SCI according to the AIS classification [25]; (iii) a stable SCI condition (i.e., at least three months after the injury); and (iv) absence of severe cognitive impairment (MoCA > 20). Exclusion criteria were as follows: (i) presence of disabling sensory alterations, including severe visual and hearing loss; (ii) active epilepsy with frequent seizures; (iii) concomitant medical and psychiatric illness that could potentially interfere with the innovative rehab pathway. Additionally, patients who had undergone multiple rehabilitation cycles were excluded. 

### 2.2. Procedures 

**Figure 1:**
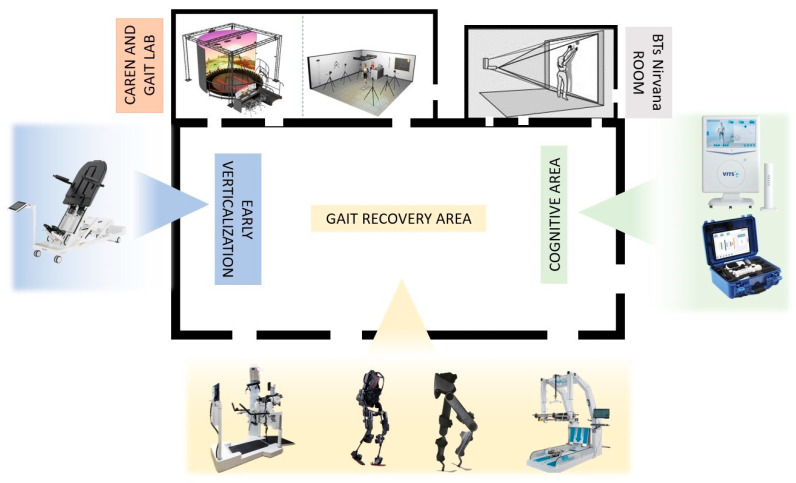
Rehabilitation environment dedicated to innovative technologies for both motor and cognitive treatment.

At admission, all patients were submitted to motor and cognitive screening tests to determine the most suitable pathway, considering the feasibility of engaging in robotic and VR rehabilitation. The treatment regimen included traditional physiotherapy sessions, speech therapy, psychological support, neuro-sexual counseling, and occupational therapy in a dedicated demotics environment, integrated with physical therapy incorporating focal vibration, robotic devices, advanced cognitive rehabilitation, and telerehabilitation in the weeks post-discharge (see Figure 1). 

### 2.3. Description of the Innovative Pathway

SCI involves complex clinical, rehabilitative, psychological, relational, and social welfare needs. Therefore, patients with SCI require a multidisciplinary approach with an individualized and specialized path. After the acute phase and medical/clinical stability, SCI patients can be managed in our Research Institute, which is a well-recognized neurorehabilitation facility, and may attend the “Robotic Neurorehabilitation” ward.

To attend the ward, patients should not have contraindications for the use of innovative technologies, including robotics. The steps of the rehabilitation path are as follows.

(A) Evaluation at the admission

At admission, evaluation by a qualified team—physician, neurologist, psychologist, speech therapist, physiotherapist, occupational therapist, and robotics nurse—guarantees suitability. The physiatrist assessment delves into the functional impact of SCI, forming the basis for an individualized rehabilitation plan. Simultaneously, the neurologist assesses neurological deficits, providing insights into sensory and motor functions critical for understanding the extent of the injury. Physiotherapists conduct a thorough physical assessment, guiding the development of tailored interventions to enhance mobility, strength, and overall physical function through subjective and objective tools. In particular, they used standardized scales such as Fugl-Meyer-FMA [26]. The Fugl–Meyer Assessment is a clinical tool for evaluating motor function, balance, and sensation in individuals recovering from stroke. It uses a scale from 0 to 2 to assess specific tasks, providing a numerical score to gauge the impairment level and track rehabilitation progress. The evaluation includes instrumented gait analysis, defined as the set of procedures that are needed to observe, record, analyze, and interpret human locomotion. The “Movement Laboratory” includes the BTS Gaitlab (BTS Bioengineering, Milan, Italy), which consists of a complete laboratory for motion analysis inside the clinic, through a complex motion capture system. It includes an optoelectronic system detecting the exact position of the markers placed on the patient’s body, and appropriate software calculates kinematic and kinetic parameters and electromyographic activity [27,28]. 

Speech therapists focus on evaluating and addressing any communication and swallowing difficulties that may arise from the SCI (especially in patients with lesions involving up to C5), ensuring comprehensive care. Occupational therapists play a crucial role in evaluating the patient’s ability to engage in daily activities. Their assessment considers factors such as fine motor skills, activities of daily living, and environmental adaptations. This information guides the development of interventions aimed at maximizing the patient’s independence and quality of life. The neuropsychological assessment, conducted by a psychologist, explores cognitive and emotional aspects, contributing to a more comprehensive understanding of the patient’s psychological well-being. Psychologists also formulate personalized psychotherapeutic plans to address emotional challenges and enhance coping strategies.

(B) Rehabilitation Plan

The collaborative efforts of our multidisciplinary team culminate in the development of an Individual Rehabilitation Plan (IRP), meticulously crafted in alignment with the International Classification of Functioning, Disability, and Health (ICF) model [29]. This personalized plan acts as a dynamic roadmap, steering interventions within the Robotic and Behavioral Neurorehabilitation Service [30]. It incorporates a diverse range of therapies, including robotic interventions, advanced cognitive rehabilitation, and VR interventions (see Table 1).

The IRP is not static; rather, it undergoes continuous reassessment and fine-tuning to ensure that it evolves with the changing needs of the patient throughout the rehabilitation journey. This holistic approach enables us to deliver comprehensive care, addressing not only the physical aspects of recovery but also attending to the cognitive, communicative, and occupational dimensions of the patient’s well-being. The adaptability of our rehabilitation plan reflects the importance of a patient-centered approach and evolving care that optimally contributes to the overall recovery and enhanced quality of life.

(C) Rehabilitation protocol

The rehabilitation program is strategically designed to incorporate the following components on a weekly basis, with each session lasting 1 h:

- A total of 5 to 7 physiotherapy sessions tailored to individual needs.
- A total of 6 to 10 robotic treatments per week, meticulously customized according to the patient’s specific physical requirements.
- A total of 3 psychological sessions weekly, comprising a supportive interview and two cognitive treatments employing VR or other innovative tools, targeting specific areas for improvement.
- A total of 2 to 5 speech therapy sessions every week.
- A total of 3 to 5 occupational therapy sessions per week.
- A total of 1 neuro-sexual consultation per week, readily available upon request.

The duration of hospitalization ranges from 2 to 6 months, primarily determined by the severity of the SCI. Guided by the expertise of the robotic neurorehabilitation specialist, the use of innovative devices is prescribed, considering contraindications for intensive applications of robotics and VR. This comprehensive approach ensures a personalized and dynamic rehabilitation plan, addressing the diverse needs of patients while optimizing the use of cutting-edge technologies.

Thus, during hospitalization, patients undergo intensive neurorehabilitation six days a week, with each day featuring a comprehensive schedule of five to seven training sessions incorporating the various types of interventions. 

In the pre-discharge phase, special emphasis is placed on social reintegration. Occupational therapy takes center stage, conducted in a home automation environment, domotics [22]. This tailored approach aims to enhance daily living skills and facilitate a smoother transition back into the community.

(D) End of hospitalization

At the end of hospitalization, rehabilitation and care services extend beyond inpatient management, with the Day Hospital (DH) offering neuromotor, cognitive, and speech therapy using rehabilitation technologies. After DH, patients can access outpatient services (with a focus on robotics) or telemedicine, ensuring continuity of care based on individual needs. 

Telemedicine monitoring is also introduced as part of the pre- and discharge process. Telemedicine allows remote rehabilitation interventions and becomes essential for patients who live far from the main hospitals. In summary, our approach is designed to progress through various treatment modalities and prioritize the patient’s holistic recovery. The focus on individualized and specialized care extends to the promotion of global functional recovery and successful social reintegration (Table 2).

### 2.4. Outcome Measures 

In this retrospective study, although our inpatient records encompass a wide range of assessments conducted by the multidisciplinary team, our focus was strategically limited to select measures. This was performed to specifically evaluate the feasibility of our pathway concerning lower limb function, cognitive abilities, emotional well-being, and quality of life. To measure improvements in lower limb strength and function, we utilized the Fugl–Meyer Assessment as a comprehensive measure [26]. To monitor mood changes, we used the Beck Depression Inventory (BDI) [31], considering the common occurrence of depressive symptoms in spinal cord injury patients. For an overall assessment of cognitive function, we employed the Montreal Cognitive Assessment (MoCA) [32], providing insights into overall cognitive performance. Additionally, we assessed patient satisfaction using the Short Form of the Patient Satisfaction Questionnaire (PSQ) [33], offering insights into the subjective evaluation of the intervention. Both baseline and post-intervention assessments were conducted, and the data were collected retrospectively for a comprehensive analysis of the outcomes throughout the intervention. Finally, to assess the quality of life, we used the Short Form-12 Health Survey (SF-12), which allows for the evaluation of total quality of life as well as perceived physical and mental well-being [34]. 

### 2.5. Statistical Analysis

The medical records of 210 SCI patients who had been treated in our Rehab Unit were examined. The final sample consisted of 42 patients. Data were entered and analyzed using GraphPad Prism 9 (RITME Corp, CA, USA). The significance level of the statistical tests was established with *p* < 0.05. Descriptive statistics were analyzed and expressed as mean ± standard deviation or as median ± first-third quartile for continuous variables, as appropriate; frequencies (%) were used for categorical variables. Nonparametric statistical tools were used to analyze the data, as the Kolmogorov–Smirnov results indicated that the target variables were not normally distributed. Therefore, we used the Wilcoxon and Mann–Whitney tests for within-group and between-group comparisons, respectively, corrected for multiple comparisons.

## 3. Results

**Figure 2:**
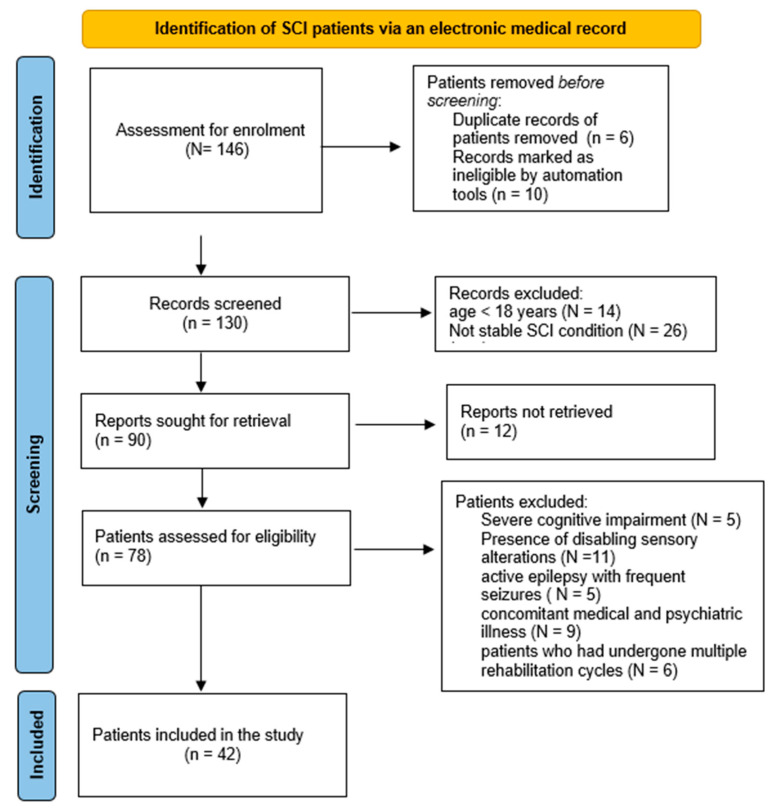
Flow chart.

All enrolled participants successfully completed the study, reporting no adverse effects. The study comprised a sample of 42 participants, and their demographic and clinical characteristics are comprehensively detailed in Table 3. It is noteworthy to provide context to the sample size, as the selection process initially involved 146 potential participants. After meticulous screening, including the removal of duplicates and incomplete data, the cohort was refined to 78 individuals. Subsequently, the inclusion criteria were applied, leading to the final inclusion of 42 participants. Figure 2 presents a flowchart showing the selection process of patients. 

The application of the Wilcoxon signed-rank test (refer to Table 4) uncovered statistically significant differences between baseline (T0) and post-intervention (T1) evaluations across all assessed domains.

Upon closer examination of pre–post comparisons, a notable and statistically significant enhancement was observed in lower limb function (*p* < 0.001), overall cognitive functioning (*p* < 0.001), and perceived quality of life, both physically and mentally (*p* < 0.001). Additionally, a significant reduction in depressive symptoms was identified (*p* < 0.001).

The study’s outcomes further highlighted a substantial level of patient satisfaction across diverse subscales. Specifically, the General Satisfaction scale achieved a score of 7 ± 2 (with a cut-off > 5 and a maximum score of 10). Technical Quality exhibited excellent outcomes, securing a score of 18 ± 2 (with a cut-off > 10 and a maximum score of 20). Interpersonal Manner earned a score of 8 ± 1 (with a cut-off > 5 and a maximum score of 10). Communication received a score of 6 ± 3 (with a cut-off > 5 and a maximum score of 10). Financial Aspects were well-received, attaining a score of 9 ± 1 (with a cut-off > 5 and a maximum score of 10). Time Spent with the doctor achieved a score of 7 ± 1 (with a cut-off > 5 and a maximum score of 10). Lastly, Accessibility and Convenience garnered a score of 16 ± 3 (with a cut-off > 10 and a maximum score of 20).

In addition, we conducted analyses to assess the mean change in various domains pre- and post-intervention, along with 95% confidence intervals (Table 4). Notably, the MoCA score exhibited a mean change of 2.5 (95% CI: 1.66, 3.34), indicating a significant improvement in overall cognitive functioning. The BDI score demonstrated a mean change of −3.8 (95% CI: −5.54, −2.06), reflecting a substantial reduction in depressive symptoms. Similarly, the SF−12 Total score displayed a mean change of 5.6 (95% CI: 4.15, 7.05), underlining improvements in overall perceived quality of life. The SF-12 Mental Health score showed a mean change of 3.6 (95% CI: 2.54, 4.66), signifying enhanced mental well-being. Additionally, the SF-12 Physical Health score exhibited a mean change of 4.1 (95% CI: 1.08, 7.12), indicating positive changes in physical health. The FMA score demonstrated a mean change of 6.2 (95% CI: 1.79, 10.61), highlighting improvements in lower limb function.

## 4. Discussion

This study aimed to assess the feasibility and potential efficacy of a comprehensive pathway for SCI, integrating traditional and technological approaches. The results demonstrated significant improvements in lower limb function, cognitive domains, quality of life, and a reduction in depressive states, consistent with findings from various studies [35,36,37,38]. 

### 4.1. Motor Outcome

The use of robotic systems and VR aligns with motor learning principles, fostering better recovery through high-intensity and task-oriented exercises [39,40]. Innovative devices are meticulously designed to streamline rehabilitation processes, notably evident in the significant enhancement observed in lower limb muscle strength as indicated by the Fugl–Meyer Assessment [19]. The implementation of these devices contributes to improved kinematic reproducibility during lower limb movements or gait cycles. Our study revealed substantial advancements in balance and the maintenance of muscle tone, aligning with the objective of promoting overall lower limb functionality [23].

After a SCI, muscle force in the lower limbs is likely to be insufficient to support the body during walking [40]. In this sense, robotic devices enable the unloading and reloading of the body, which are essential elements in inducing training effects on the spinal locomotor centers. The afferent inputs provided by the contact between the foot and the ground are crucial for activating the neural circuits underlying locomotion [40,41]. In addition, robotics also play a role in the modulation of spasticity. During daily locomotor training, the partial unloading of body weight, combined with rhythmic cyclic movements, facilitates stable stepping and reduces inappropriate activation of the tibialis anterior. This aspect highlights the significance of movement awareness and quality, which is facilitated by multisensory feedback [42,43,44]. On the other hand, VR provides immediate feedback through multisensory stimulation, teaching the body and brain how to correct patients’ movements based on what they have learned (reinforcement learning). According to Scandola et al. [45], patients with SCI report improvements in both motor and cognitive functions when they are exposed to visual motor feedback provided by VR. Given that VR provides input from higher-level networks to basic ones, it affects motor programming and influences visuomotor and sensorimotor areas and peripheral structures.

### 4.2. Cognitive Domains

The integration of robotics and VR into our interventions may promote cognitive function, as shown in other studies [21,22,46,47]. In our sample, personalized and integrated methodologies boosted cognitive abilities, as demonstrated by the results of the Montreal Cognitive Assessment (MoCA). Furthermore, our results highlighted how important it is to pay attention to the cognitive components of the SCI patient, which are often neglected in the healthcare service. The few published studies on this topic differ in terms of design, types of cognitive tests used, and specific cognitive domains assessed, including attention, concentration, executive function, memory, processing speed, and cognitive flexibility, as well as depression and anxiety [21,22,47]. However, a recent review [48] showed significant deterioration in one or more of these cognitive domains following SCI. According to our results, some authors [49,50,51] found that patients with SCI are approximately 13 times more likely to experience cognitive impairments than their able-bodied counterparts.

This highlights the need to incorporate cognitive and emotional assessments of the rehabilitation framework, ensuring a more holistic and tailored approach to addressing the various challenges faced by people with SCI.

Cognitive impairment is a significant complication in the SCI population, manifested by various alterations in cognitive functions. Some authors, such as Murray et al. [52], Molina et al. [53], and Chiaravalloti et al. [54,55], have helped highlight these cognitive impairments, including decreased attention, impaired visuospatial perception, reduced problem-solving ability, reduced processing speed, and decreased memory and learning abilities as well as the risk of aggressive behavior. This may lead to a negative impact on patients’ quality of life and new hospital admissions [52,53,54,55].

Several factors have been proposed as potential contributors to these cognitive impairments, including concomitant traumatic brain injury, hypoxia and anoxia, cardiovascular and cerebrovascular dysfunction, sleep disorders such as obstructive sleep apnea, body temperature dysregulation, and substance and alcohol abuse. The severity and incidence of this disorder have been studied, with evidence suggesting it will worsen over time, especially in the chronic phase. Molina et al. [53] reported that the use of neuroactive medications for mood and pain disorders can negatively impact cognition.

Furthermore, another factor appears to be related to the anatomical level of SCI, indicating that patients with SCI at or above the T1 level show worse performance in cognitive tasks. Finally, post-SCI pain has been identified as negatively impacting cognitive function and quality of life. 

Chiaravalloti et al. [54,55] highlight the importance of accurate assessment of cognitive function, advocating detailed neuropsychological assessments [49]. 

In line with these authors, we considered it important to introduce an accurate cognitive assessment and rehabilitation of the cognitive components into our path. As highlighted in other pathologies, VR has the potential to help enhance cognitive components by promoting neuroplasticity processes. Through the feeling of immersion in the training context and the increase in feedback that allows awareness of the results and movement of one’s body, VR has achieved several positive results in patient recovery, as observed in our sample.

### 4.3. Mood and Depression

The BDI score showed a mean change of −3.8 (95% CI: −5.54, −2.06), indicating a significant decrease in depressive symptoms. We believe that our personalized rehabilitation program contributed to this substantial reduction in depressive symptoms. This result is highly significant because SCI can have a substantial impact on mental health, particularly in terms of depression [56]. Research indicates that approximately one-quarter to one-third of individuals with SCI experience significant levels of depression after injury [57,58]. Depression persists for many years post-discharge and is associated with a higher incidence of secondary health complications such as pressure sores and urinary tract infections [57,58]. Suicide rates among individuals with SCI remain more than three times higher than the general population, underscoring the need for psychological interventions throughout the entire lifespan of individuals with SCI [59]. 

In line with these concerns, we have observed that the combined use of innovative rehabilitation with other interventions can contribute to a reduction in depressive symptoms. Indeed, the combination of robot-assisted therapy and VR has been shown to stand out as an innovative approach with substantial benefits for improving mood and alleviating depressive symptoms in patients with different neurological disorders [60]. While studies have traditionally focused on outcomes related to motor improvement and change in functional status, robotics and VR, alone or in combination, have shown a broader impact on the mental health and psychological well-being of individuals. Then, our findings are in line with some research, which goes beyond motor-related findings, highlighting the positive changes in depression symptoms [61]. Indeed, other studies by our group have highlighted that the introduction of innovative rehabilitation can allow an increase in perceived well-being, better coping strategies, and better quality of life [7,9,12]. 

### 4.4. Motivation, Patient Satisfaction, Quality of Life

Moreover, the results of the Short Form-12 Health Survey (SF-12) reflected positive outcomes in enhancing patients’ perceived quality of life. This positive impact was particularly observed in both the physical and mental dimensions, signifying the comprehensive influence of our interventions. Thus, despite the potential benefits of rehabilitation devices, our study emphasized the fundamental importance of considering patient perceptions and adherence, often overlooked in the literature. Investigating users’ perceptions of neurorehabilitation is pivotal, as patients’ motivation significantly predicts long-term changes in quality of life and rehabilitation outcomes [7]. Previous research confirmed that the combined use of conventional methods and technological devices could maximize rehabilitation effectiveness [60,62,63,64]. Furthermore, our findings highlighted the satisfaction of patients with the multidisciplinary rehabilitation treatment and their active involvement in the recovery process. It is noteworthy that personalizing the treatment by incorporating innovative devices played a crucial role in actively engaging patients and ensuring the success of the rehabilitation pathway [65]. Our study attested to the success of this approach in increasing patient motivation, fostering active participation, and elevating the overall quality of interventions. The personalized rehabilitation paths, guided by the combination of robotics and VR, notably contributed to improved patient satisfaction, as evidenced by the increased scores in various satisfaction scales. Motivation is a key element in the rehabilitation process, with implications at the brain’s mesolimbic level. In humans, the reward-motivated behavior circuit is primarily controlled by the medial and lateral pre-frontal cortex, which integrates motivation and cognitive control during decision making [66]. This circuit also involves the dorsolateral pre-frontal cortex, thalamus, and insula, which are responsible for motivated behavior and the subsequent control of motor actions [67]. In summary, motivation not only increases adherence to treatment but also promotes greater involvement of brain areas, leading to neuroplasticity.

On the contrary, it has been shown that patients who perceive systems as useless and lack motivation encounter more difficulty in their use and exhibit reduced therapeutic adherence [68]. Incorporating various devices in our innovative rehabilitation path allowed us to provide personalized and patient-centric care, receiving positive feedback from participants. In line with our findings, Resquin et al. noticed that patients found innovative rehabilitation attractive, embracing an active attitude without feeling pressured or stressed [69]. Additionally, Pei et al. showed that high usability scores in healthy subjects and stroke patients undergoing robot-assisted therapy underscored the significance of user-friendly technologies [70]. 

Moreover, our findings indicated high levels of patient satisfaction across various dimensions. Participants responded positively to the use of innovative rehabilitation interventions, recognizing supportive interactions with healthcare professionals. Satisfactory communication practices, including positive feedback on financial aspects, were noted. Another point that should be considered for the success of our pathway is the presence of a skilled equipe of healthcare professionals. The therapists played a crucial role in ensuring an adequate response to the needs of the patients, highlighting collaborative efforts from professionals with diverse expertise contributing to the success of the patient-centric and innovative rehabilitation pathway. Thus, the multidisciplinary team, through complementary abilities and knowledge, can encounter different patient needs, including motor, emotional, social, and cognitive. Then, a global patient care strategy, encompassing all facets of the patient’s well-being, is vital for achieving positive long-term outcomes [70]. 

### 4.5. Limits to the Use of Innovative Devices

While there is compelling evidence supporting the potential benefits of incorporating robotic devices and VR into neurorehabilitation, recent reviews, such as the one conducted by Resquín et al. [69], highlighted certain limitations associated with the use of these systems. In particular, the challenges arise from the integration between the patient and the machine, along with variations in clinical conditions among patients. In our retrospective analysis, some participants were excluded due to difficulties during experimental sessions, raising awareness of potential limitations in widespread applicability. This influenced their perception of the usability of the instruments. As emphasized by Resquìn et al. [69], careful attention must be given to patient inclusion criteria for innovative treatment, such as robotic devices, a point also endorsed by Huang et al. [50]. Several studies [71,72,73,74,75,76] have indicated that robotics appears to be more beneficial for individuals with moderate to severe deficits. In contrast, patients with better motor function may not derive additional benefits from training with innovative devices compared to conventional methods. These insights underscore the importance of refining patient selection criteria to optimize the effectiveness and appropriateness of robotic rehabilitation interventions. 

### 4.6. Study Limitations

This retrospective study had several limitations that need to be considered in interpreting the results. Firstly, the absence of a randomized control group might impact the ability to establish causal relationships between the variables considered and the observed outcomes that could be related somehow to a spontaneous recovery. However, our aim was not to investigate the efficacy of our innovative pathway but rather its feasibility and the potential beneficial role in SCI patients. Further larger sample studies with a control group receiving conventional therapy are needed to assess the efficacy of our promising protocol.

Additionally, the inherent risk of selection bias in retrospective studies, relying on pre-existing data, could introduce a certain degree of data-selection bias. Data collection based on individuals’ memories may contribute to the risk of memory bias, involving potential recall errors or information incompleteness. Variability and quality fluctuations in the data over time add an element of uncertainty to our analysis. Lastly, the difficulty in fully controlling confounding variables and the challenges in establishing a causal relationship are intrinsic aspects of studies of this nature. However, the aim of the study was the feasibility of this dedicated, innovative pathway, and we did not consider other outcome measures sensible in more specifically detecting motor and cognitive changes due to treatment. 

## 5. Conclusions

In conclusion, this retrospective study provides valuable information on the potential benefits of an innovative rehabilitation pathway for patients with SCI. Our results suggested that a multidisciplinary approach improved motor function, cognitive functions, and psychological well-being as well as quality of life in patients with SCI. However, larger sample studies with a control group and long-term outcomes are needed to confirm these encouraging results.

## Figures and Tables

**Table 1:** Shows robotic and virtual reality devices for both motor and cognitive rehabilitation in SCI patients.

Type of Intervention	Device/Tool	Outcome	Description
Robotic	Erigo (Hocoma, Volketswil, Switzerland)	Verticalization	Erigo consists of a robotic oscillating table that allows early and progressive robotic verticalization in the acute post-SCI phases and is combined with allowing cyclical leg movement. The tilting table, from 45° to 90°, can be adapted by therapists according to the patient’s needs, and it is also possible to customize the step speed. In addition to verticalization, the device helps to improve the cardiovascular system by activating the muscles and promoting venous return.
	Lokomat (Hocoma, Volketswil, Switzerland)	Gait training	The Lokomat is a robotic exoskeleton equipped with a treadmill and a weight relief system. It is a tethered exoskeleton with powered orthoses at the hip and knee, passive ankle control during the swing phase, and variable levels of assistance. It can be fitted with a VR screen (Lokomat Pro) to enhance patients’ motivation during training. Additionally, the Free D model allows pelvic movement, simulating physiological human gait.
	Ekso-GT/-NR (Ekso Bionics, San Rafael, CA, USA)	Gait training	The Ekso, in contrast, is an untethered exoskeleton designed as a wearable powered orthosis at the hip and knee joints. Patient-initiated walking is facilitated through lateral weight-shifting movements. This untethered design allows flexibility in mobility. The Ekso provides adaptable assistance based on individual patient needs, accommodating unilateral or bilateral support. It is specifically intended for individuals with functional upper extremity strength and spinal cord injury levels T4-L5, as well as C7-T3 (AIS D), making it a versatile solution for diverse rehabilitation scenarios.
	Indego (Ekso Bionics, San Rafael, CA, USA)	Gait training	The Indego, a hip–knee exoskeleton, is a dynamic and powered wearable device designed specifically for gait training. Engineered for individuals with spinal cord injuries at C7 and lower levels within rehabilitation facilities, and T3 and lower levels for home and community use, the Indego provides a versatile solution for diverse settings. Activation of walking is initiated by the individual’s intentional center of pressure (COP) movement, either in the anterior direction to commence walking, sit–stand maneuvers, or in the posterior direction to initiate stopping or stand–sit transitions. This sophisticated exoskeleton thus responds to the user’s intentional cues, promoting an intuitive and personalized gait-training experience.
	G-EO System (Reha Technology, Olten, Switzerland)	Gait training	Gait training with the G-EO System involves a robotic end effector system that replicates the movements of walking, as well as ascending and descending stairs. The patient’s feet are securely fastened to platforms capable of multidirectional movements, facilitated by six engines aiding in various directions—upwards, downwards, forwards, and backwards. This innovative system offers a comprehensive approach to gait simulation, promoting a dynamic and effective training experience for patients.
Virtual reality	BTs Nirvana (BTS Bioengineering, Milano, Italy)	Motor and cognitive functions	BTS-Nirvana is a semi-immersive virtual reality (VR) system composed of computer software, two markerless optoelectronic infrared sensors, a video camera, and a projector connected to a large screen. Users interact fully with the virtual environment through their movements, effortlessly captured by the infrared sensors. The proposed activities include exercises that require patients to perform specific actions, such as reaching, touching, or grabbing projected objects, as well as interacting with projected images on the floor, such as balls, providing dual-task activities that involve both motor and cognitive aspects.
	VRRS (Khymeia, Padua, Italy)	Balance, language, and cognitive functions	The Virtual Reality Rehabilitation System (VRRS) is designed around a central hub, connectable via USB, accompanied by a set of specialized peripherals meticulously synchronized and seamlessly integrated with the system. VRRS is outfitted with exercise modules catering to cognitive, language, postural, and motor rehabilitation. Therapists have the capability to select and incorporate virtual exercises into the rehabilitation program, shaping the difficulty level in correlation to the timing of execution and the nature of the activity. This adaptable and comprehensive system allows for tailored rehabilitation programs to meet individual patient needs.
	CAREN (Motek, Amsterdam, The Netherlands)	Gait training, balance, and cognitive functions	The Computer Assisted Rehabilitation Environment (CAREN) is comprised of an electro-hydraulic 2 m diameter motion platform, offering manipulation across 6 degrees of freedom. During each session, the patient stands on this dynamic platform, featuring force plates beneath a double-banded treadmill capable of reaching speeds of up to 5 m/s. The platform’s movement is either user-driven or preprogrammed, synchronized with function curves defining specific pathways within the virtual environment. Additionally, the device incorporates a 180° screen, providing varying levels of virtual reality immersion, ranging from flat video and dual-channel audio to a fully enveloping 360-surround sound dome enclosure.
Telerehabilitation	VRRS-HomeKit (Khymeia, Padua, Italy)	Motor functions (lower and upper limbs, balance) and cognitive functions	The Virtual Reality Rehabilitation System (VRRS) HomeKit is a portable device featuring a tablet that facilitates virtual exercises for patients. Interaction occurs with 2D scenarios and objects using the touchscreen or various sensors. For instance, the K-wand is employed for movement tracking and orientation, manipulated by the patient during catching and reaching exercises for upper limbs. Additionally, a pair of K-sensors, comprising sensors on wearable strips of varying sizes, is utilized for conducting full-body motor tele-training activities.

**Table 2:** Strengths of the rehabilitation pathway.

Strengths of the Rehabilitation Pathway
1. Initial Objective Assessment
The rehabilitation process begins with an initial objective evaluation, using specific scales to define the patient’s global profile and define a personalized rehabilitation project.
2. In-Depth Gait Analysis
After the initial assessment, an in-depth gait analysis is conducted by using the BTS Gaitlab (e.g., optoelectronic system with markers and electromyographic probes) to objectively analyze the patient’s locomotor capabilities (kinetic, kinematic, and electromyographic parameters).
3. Individualized Rehabilitation Plan
Based on the assessments, an individualized rehabilitation plan is formulated, aligning with the International Classification of Functioning, Disability, and Health (ICF) model.
4. Multidisciplinary Rehabilitation
Rehabilitation integrates conventional treatments with innovative ones aimed at improving motor, emotional, cognitive, speech therapy, occupational, and social outcomes.
5. Integration of Robotics
Throughout the hospitalization, the integration of robotics is a pivotal strength, providing innovative interventions tailored to enhance neurorehabilitation and providing repetitive, intensive, and task-oriented training.
6. Virtual Reality Rehabilitation
Virtual reality is seamlessly incorporated into the rehabilitation program, offering advanced cognitive rehabilitation and immersive experiences for patients.
7. Pre-Domiciliation Trials with Home Automation
Starting a month before discharge, weekly pre-domiciliation trials, including home automation, are introduced to familiarize patients with daily activities.
8. Continuation through Day Hospital and Outpatient Programs
The holistic approach extends beyond hospital admission, maintaining rehabilitation through day hospital services and outpatient programs, ensuring continuous and sustained progression toward the patient’s functional recovery.
9. Telerehabilitation
Telerehabilitation is implemented as a vital component, facilitating remote interventions to support patients residing far from main hospitals and ensuring continuity of care based on their needs.

**Table 3:** Demographic and clinical characteristics of the patients.

	Patients
Number of patients	42
Age (years)	52.21 ± 15.26
Gender	
Female	25 (59.5%)
Male	17 (40.5%)
Education	-
Elementary school	1 (2.4%)
Middle school	12 (28.6%)
High school	23 (54.8%)
University	6 (14.2%)
Spinal Injury Disability (AIS)	
AIS-A patients	20 (47.6%)
AIS-B patients	22 (52.3%)
Time post-injury in months	
AIS-A patients	7 ± 2
AIS-B patients	7 ± 2

Mean ± standard deviation were used to describe continuous variables; proportions (numbers and percentages) were used to describe categorical variables. ASIA—American Spinal Injury Association, AIS—Abbreviated Injury Scale.

**Table 4:** Wilcoxon’s test of neuropsychological and lower limb functions evaluation.

	T0 Mean ± SD	T1 Mean ± SD	*p*-Value	Mean Change (95% Confidence Interval)
MoCA	22.1 ± 3.3	24.6 ± 2.8	<0.0001 *	2.5 (1.66, 3.34)
BDI	13.7 ± 7.0	9.9 ± 7.1	<0.0001 *	−3.8 (−5.54, −2.06)
SF-12 TOT	26.1 ± 6.1	31.7 ± 8.1	<0.0001 *	5.6 (4.15, 7.05)
SF-12 MENT	17.0 ± 5.5	20.6 ± 5.8	<0.0001 *	3.6 (2.54, 4.66)
SF-12 PHY	12.8 ± 3.7	16.9 ± 3.6	<0.0001 *	4.1 (1.08, 7.12)
FMA	13.5 ± 3.8	19.7 ± 6.0	<0.0001 *	6.2 (1.79, 10.61)

* Significant differences are in bold. Legend: Beck Depression Inventory (BDI); Fugl–Meyer Assessment (FMA); Montreal Cognitive Assessment (MoCA); Standard deviation (SD); Short Form-12 Health Survey Total (SF-12 TOT); Short Form-12 Health Survey Mental Health (SF-12 Mental Health); Short Form-12 Health Survey Physical (SF-12 Physical).

## Data Availability

Data are available on request from the corresponding author. The data are not publicly available due to privacy reasons.
